# Comparative Expression Profiling Reveals Molecular Markers Involved in the Progression of Cutaneous Melanoma towards Metastasis

**DOI:** 10.3390/ijms24076565

**Published:** 2023-03-31

**Authors:** Andreea D. Lazăr, Sorina Dinescu, Lea Sleiman, Adrian V. Dumitru, Mariana Costache, Marieta Costache

**Affiliations:** 1 Department of Biochemistry and Molecular Biology, University of Bucharest, 050095 Bucharest, Romania; andreea.lazar@bio.unibuc.ro (A.D.L.); sleiman.lea@s.bio.unibuc.ro (L.S.); marieta.costache@bio.unibuc.ro (M.C.); 2 Research Institute of the University of Bucharest (ICUB), 050663 Bucharest, Romania; 3 Emergency University Hospital of Bucharest (SUUB), 050098 Bucharest, Romania; dr.adriandumitru@yahoo.com (A.V.D.);; 4 Department of Pathology, University of Medicine and Pharmacy Carol Davila Bucharest, 050474 Bucharest, Romania

**Keywords:** cutaneous melanoma, lymph node metastasis, brain metastasis, EGFR, MMP2, IL1B, MAGEC1, microRNAs, FFPE samples

## Abstract

Cutaneous melanoma is one of the most aggressive types of cancer and often proves fatal in metastatic stages. Few treatment options are available, and its global incidence is quickly increasing. In order to gain an improved understanding of the molecular features regarding melanoma progression, we have compared gene and small non-coding RNA expression profiles from cell lines derived from primary melanoma (MelJuSo), lymph node metastasis (MNT-1) and brain metastasis (VMM1), representing distinct stages of malignant progression. Our preliminary results highlighted the aberrant regulation of molecular markers involved in several processes that aid melanoma progression and metastasis development, including extracellular matrix remodeling, migratory potential and angiogenesis. Moreover, bioinformatic analysis revealed potential targets of the microRNAs of interest. Confocal microscopy and immunohistochemistry analysis were used for validation at the protein level. Exploring the molecular landscape of melanoma may contribute to the achievement of future efficient targeted therapy, as well as better prevention, diagnosis and clinical management.

## 1. Introduction

Cutaneous melanoma is among the most aggressive types of cancer, with metastasis representing the leading cause of melanoma-related mortality. Current therapies are rarely curative for metastatic melanoma, and despite existing progress in the development of new therapeutic approaches, this type of skin cancer continues to be lethal. Thus, there is an urgent need to identify more effective preventive and therapeutic targets, and this can be achieved by improving our understanding of the molecular mechanisms involved in the metastatic progression of melanoma [1].

Global expression profiling has proven to be a significant tool in helping to uncover the molecular basis of cancer, generating new insights into melanoma development. For instance, genomic analyses have established the molecular classification of melanoma based on the most frequent driver oncogenes (*BRAF*, *NRAS*, *KIT*) and have also revealed a long list of rare events, whose contribution to melanoma progression toward metastasis remain mostly unclear [2]. One example would be the up-regulation of *BIRC5*-encoded survivin, which promotes melanoma metastasis through the Akt-dependent up-regulation of α5 integrin [3]. Another example would be *NOL7*, a recently identified gene with an oncogenic function in melanoma growth and evolution, found to play a role through the HIF-1α/NOL7/HRas/PI3K/AKT/ERK axis [4].

Apart from genes, numerous microRNA molecules (miRNAs/miR) have been identified and are expected to be targets for preventing melanoma metastasis. Substantial evidence points to the involvement of miRNAs in each stage of melanoma progression [5,6]. For instance, let-7a, miR-21, miR-30d/30b and miR-214 can influence ECM remodeling, by targeting integrins, increasing matrix metalloproteinases (MMPs) and diminishing the metallopeptidase inhibitors (TIMPs), thus promoting ECM degradation and decreasing tumor cell-matrix adhesion [7].

Despite this significant accumulation of knowledge, it has yet to yield considerable clinical benefits in terms of improved treatment options or overall patient survival [8,9]. As such, it remains an active field of research for scientists in pursuit of a complete overview of melanoma metastasis. This study aimed to identify differentially expressed genes and miRNAs between primary and metastatic melanoma samples, which could serve as potential biomarkers of prognostic value and therapeutic targets for improved clinical cancer diagnosis and management.

## 2. Results

### 2.1. Comparative Expression Profiling of Primary and Metastatic Melanoma Cells

Primary melanoma (PM), lymph node metastasis (LNM) and brain metastasis (BM) cell lines (MelJuSo, MNT-1 and VMM1, respectively) were examined at the gene and miRNA level using qPCR array with the intention of highlighting the differences between these representative cells for distinct tumor stages of cancer. A small number of genes, which registered the most significant aberrant regulation, were also evaluated at the protein level for confirmation, through the use of immunostaining coupled with confocal microscopy.

#### 2.1.1. Gene Expression Profiling

The differentially expressed genes between melanoma metastatic cell lines and PM cells were screened using qPCR array and the corresponding Analysis Tool (see the Materials and Methods section for a detailed description). Heatmaps were generated to provide a general view of the aberrant expression of the 252 genes that were evaluated (Supplementary Figure S1A).

**Figure 1:**
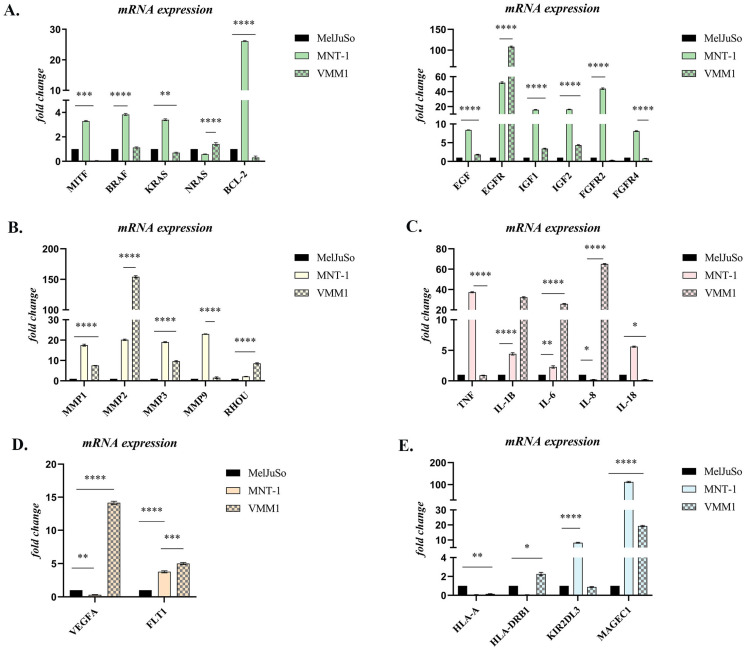
Differential expression of genes involved in: (**A**) Survival and proliferation; (**B**) Migration and invasion; (**C**) Inflammation; (**D**) Angiogenesis; (**E**) Immune response modulation. Statistical significance: “*” symbol was used for statistical comparisons between cell lines derived from PM (MelJuSo), LNM (MNT-1) and BM (VMM1); * *p* < 0.05; ** *p* < 0.01, *** *p* < 0.001, and **** *p* < 0.0001 (n = 3, biological replicates).

Based on the GeneCopoeia Data Analysis, several genes mostly associated with cell proliferation, survival, invasion, inflammation, angiogenesis and immune response modulation were highlighted, and a graphical representation of these genes is shown in Figure 1. The genes selected showed a ≥2-fold difference in expression and *p* < 0.05 (for additional information see Table S1).

Gene expression analysis identified around 200 genes that showed significant differential expression, with the most statistically significant ones being graphically represented in Figure 1. Most differences reflected increased gene expression in metastases relative to primary samples. Reduced gene expression in metastatic cells was less frequent and less dramatic. Interestingly, most of the genes that were up-regulated in the LNM cell line (MNT-1), were down-regulated in BM cells (VMM1), or vice versa, with a few notable exceptions, including *EGFR*, *MMP2*, *IL1B* and *MAGEC1*. Genes significantly increased or decreased in metastases were active in cell cycle regulation, proliferation, apoptosis, cell motility, inflammation, angiogenesis and immune response modulation (Figure 1).

In the case of *MITF*, a marker proclaimed as a “phenotype switch” in melanoma, a statistically significant increase in expression was observed in the MNT-1 (*p* < 0.0001, approximately 3-fold) compared to MelJuSo, while in VMM1 it was significantly down-regulated (*p* < 0.001). A statistically high expression of *BRAF*, *NRAS* and *KRAS*, important genes involved in the MAPK signaling pathway, was observed in the case of MNT-1 and VMM1 cell lines (*p* < 0.0001), with significant differences between the two types of metastases being registered for *RAS* oncogenes (*p* < 0.0001), which indicates the activation of this cascade and the subsequent promotion of cell survival and proliferation. Additionally, tyrosine-kinase receptors *EGFR*, *FGFR2* and *FGFR4* were significantly up-regulated in metastatic cells, with *EGFR* being highly expressed in both (50-fold change in MNT-1 and 110-fold change in VMM1), while FGF receptors registered a low expression in VMM1 (*p* < 0.01). Growth factors *EGF*, *IGF1* and *IGF2* were significantly overexpressed in the metastatic cell lines compared to primary cells (*p* < 0.0001), with a higher expression in MNT-1. Similarly, the expression of anti-apoptotic factor *BCL-2* was significantly higher in the LNM cell line compared to primary stage melanoma MelJuSo (~26 times higher), although it registered a significant decrease in VMM1 (*p* < 0.0001) (Figure 1A).

Matrix metalloproteinases *MMP1*, *MMP2*, *MMP3* and *MMP9*, which are involved in extracellular matrix (ECM) remodeling and promotion of tumor cell migration and invasion, were also significantly increased (*p* < 0.0001) in metastatic cell lines compared to PM cells. Of note is the dramatic overexpression of *MMP2* in VMM1 (approximately 150-fold change). Interestingly, overexpression of *RHOU*, a Rho GTPase involved in cell motility through lamellipodia formation, was observed in metastatic cells (*p* < 0.0001, eight times higher in VMM1 and two times higher in MNT-1) when compared to primary tumor cells (Figure 1B).

Overexpression of inflammatory markers *TNF*, *IL1B*, *IL-6*, *IL-8* and *IL-18* is highlighted in Figure 1C. TNF registered an increased expression in MNT-1 (~38 times higher), but remained unchanged in VMM1, while *IL-8* was significantly up-regulated in distant metastatic cells (*p* < 0.0001, 65-fold change) and down-regulated in the LNM cell line (*p* < 0.05). Up-regulation of *IL1B* and *IL-6* was found to be statistically significant (*p* < 0.0001) in both metastatic cell lines, with an approximative 4-fold difference in MNT-1 and 30-fold change in VMM1. Meanwhile, overexpression of *IL-18* was observed in the case of MNT-1, compared to MelJuSo (*p* < 0.0001), with a lower expression being registered in VMM1 (*p* < 0.05).

Angiogenesis markers *VEGFA* and *FLT1* (also known as *VEGFR1*) were also found to be differentially expressed in metastatic cell lines (Figure 1D). A statistically significant increase in *FLT1* gene expression was discovered in both MNT-1 and VMM1 (*p* < 0.0001), while *VEGFA* registered a high level of expression only in BM cells, being significantly down-regulated in the LNM cell line (*p* < 0.01) when compared to PM cells (MelJuSo).

Finally, differential expression of immune markers *HLA-A*, *HLA-DRB1*, *KIR2DL3* and *MAGEC1* was highlighted in Figure 1E. Slightly increased expression of *HLA-DRB1* could be observed in VMM1 (*p* < 0.05), while overexpression of *KIR2DL3* could be found in MNT-1 (~8-fold change) when compared to primary tumor cells. Significantly high levels of expression were registered for *MAGEC1* in both metastasis-derived cell lines (*p* < 0.0001), with a dramatic increase in MNT-1 (115-fold change), while *HLA-A* had lower levels of expression in metastatic cells versus PM cells (*p* < 0.01).

#### 2.1.2. Protein Expression Evaluation of Select Genes in Melanoma Cells

To confirm the reliability and accuracy of the results, the differential expression of select genes (*EGFR*, *MMP2*, *IL1B* and *MAGEC1*), which may be considered metastasis drivers due to their high level of expression in both LNM and BM cell lines, was also evaluated at the protein level through immunofluorescence coupled with confocal microscopy. Images were analyzed using Nikon NIS Elements software (version 5.21 64-bit) and processed using ImageJ (version 1.x bundled with 64-bit Java 8) in order to quantify the fluorescence levels [10].

**Figure 2:**
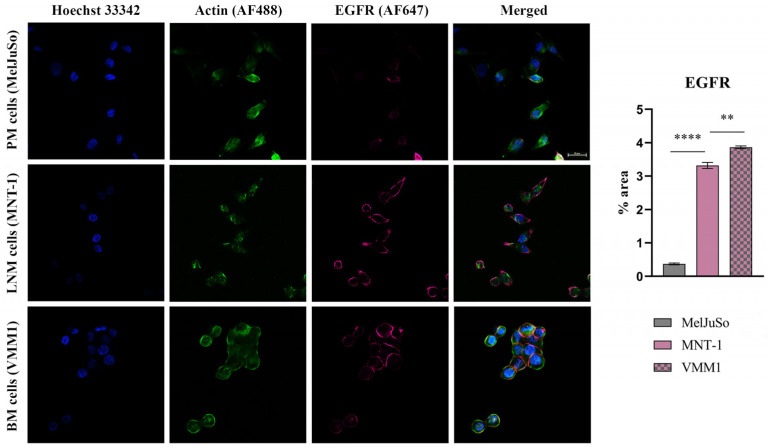
Confocal immunofluorescence microscopy images revealing EGFR protein expression in different stages of melanoma progression (scale bar 20 μm). EGFR is shown in violet (AF647), actin filaments in green (phalloidin-FITC) and cell nuclei in blue (Hoechst 33342). Quantitative measurement of fluorescence intensity was performed with ImageJ software; ** *p* < 0.01 and **** *p* < 0.0001.

**Figure 3:**
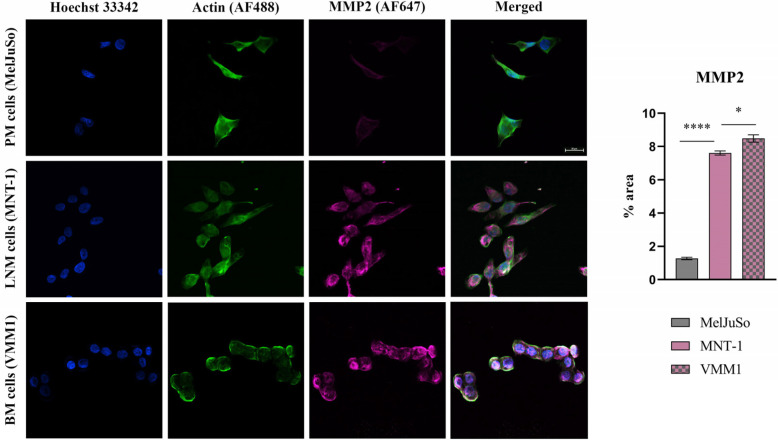
Confocal immunofluorescence microscopy images revealing the protein expression of MMP2 (violet—AF647), actin filaments (green—phalloidin-FITC) and nuclei in blue (Hoechst 33342). Graphical representation of fluorescence intensity is shown on the right side; * *p* < 0.05 and **** *p* < 0.0001.

**Figure 4:**
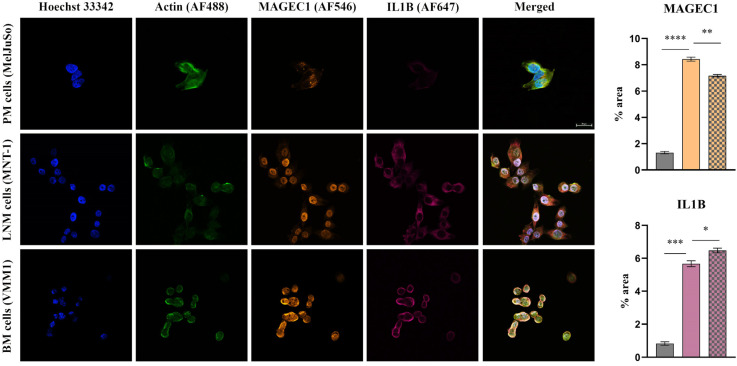
Confocal immunofluorescence microscopy images revealing the protein expression of IL1B (violet—AF647) and MAGEC1 (orange—AF546) in PM, LNM and BM cells (scale bar 20 μm). Actin filaments are displayed in green (phalloidin-FITC) and cell nuclei in blue (Hoechst 33342). Quantitative measurement of fluorescence intensity is depicted on the right (MelJuSo represented on the first column, MNT-1 on the second column and VMM1 on the third column); * *p* < 0.05; ** *p* < 0.01, *** *p* < 0.001, and **** *p* < 0.0001.

As can be observed in Figure 2, Figure 3 and Figure 4, the investigated markers had a much higher expression in metastasis-derived cells, compared to primary melanoma cells. EGFR, MMP2 and IL1B protein expression was higher in VMM1, almost four times for EGFR (*p* < 0.0001, Figure 2), eight times for MMP2 (*p* < 0.0001, Figure 3), and seven times for IL1B (*p* < 0.001, Figure 4), with slightly lower levels for MNT-1 cells, as revealed by the quantification of fluorescence intensity. Meanwhile MAGEC1 was better expressed in MNT-1 (approximately eight times higher, *p* < 0.0001), being slightly decreased in VMM1 in comparison, results which are in accordance with the gene expression analysis.

#### 2.1.3. miRNA Expression Profile and Functional Analysis

**Figure 5:**
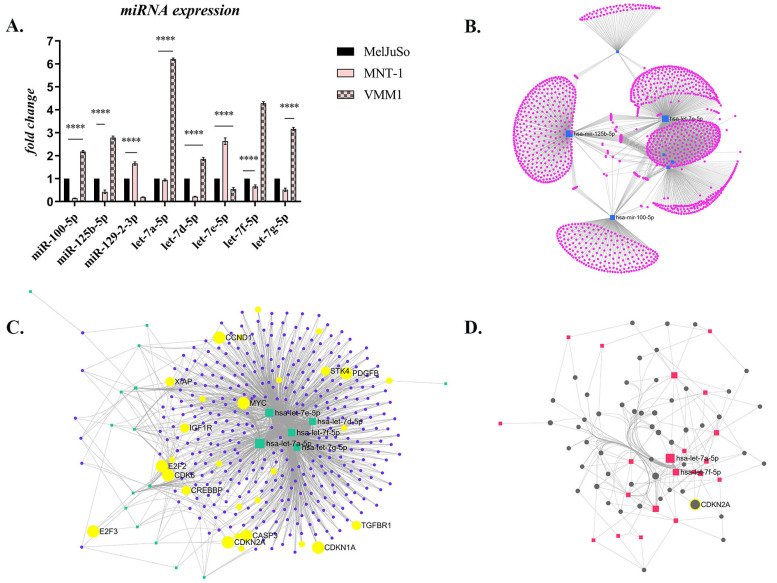
The differential expression of miRNAs in melanoma: (**A**) Selected miRNAs whose expression was notably dysregulated (**** *p* < 0.0001; n = 3, biological replicates); (**B**) Target network which shows the gene clusters (1649 genes) targeted by the miRNAs of interest (obtained with miRNet 2.0 algorithm, Degree filter, cutoff 1, layout backbone); (**C**) Target network which shows the 374 target genes, with the ones involved in several important processes for cancer being highlighted in yellow (Degree cutoff 2, layout backbone); (**D**) Minimal Network of 52 target genes, among them CDKN2A.

miRNA molecules were evaluated through the use of qPCR array and the corresponding Analysis Tool available from GeneCopoeia. The results showed that most of the evaluated miRNA molecules were down-regulated in metastatic melanoma cells compared to the primary cell line, with some notable exceptions. Additionally, differentially expressed miRNAs were also observed between the distant metastasis cells (VMM1) and the cell line derived from LNM (MNT-1) (Figure 5A; for additional information regarding the analyzed miRNAs see Figure S1B and Table S1 in Supplementary Materials).

The few notable exceptions, which were significantly overexpressed in metastatic cells compared to the primary melanoma cell line (*p* < 0.0001), were miR-100-5p, miR-125b-5p, miR-129-2-3p and some members of the let-7 family, specifically let-7a-5p, let-7d-5p, let-7e-5p, let-7f-5p and let-7g-5p (Figure 5A). Interestingly, miRNA expression also varied between the different metastatic cell lines, being diametrically opposed and statistically significant (*p* < 0.0001). Thus, miR-100, miR-125b, and several members of the let-7 family (let-7a, -7d, -7f, and -7g) showed a statistically significant increase in VMM1 compared to MelJuSo or LNM-derived cells, while the expression of miR-129 and let-7e increased significantly in MNT-1 compared to the others. The results suggest that the modulation of miRNA expression facilitates the malignant progression of cutaneous melanoma from the primary stage to metastasis.

Next, functional analysis and visual exploration of miRNA-target interactions in a network context was determined using the miRNet 2.0. platform [11]. Target genes were searched for in the miRTarBase v8.0 and miRecords databases. Initial analysis revealed 1649 genes of interest involved in multiple pathological processes, which are presented as clusters in Figure 5B. After data filtering, 374 target genes were obtained (Figure 5C) and enrichment analysis was conducted using the Hypergeometric test, and the Kyoto Encyclopedia of Genes and Genomes (KEGG) pathway revealed the involvement of these target genes in several cellular processes (Table 1), including melanoma.

The Minimum Network algorithm reduced the above number to 52 genes, highlighting common targets for the miRNAs of interest, among them CDKN2A (Figure 5D), a well-established gene involved in melanoma.

### 2.2. Validation of Select Markers on Primary and Metastatic Melanoma Patient Samples

**Figure 6:**
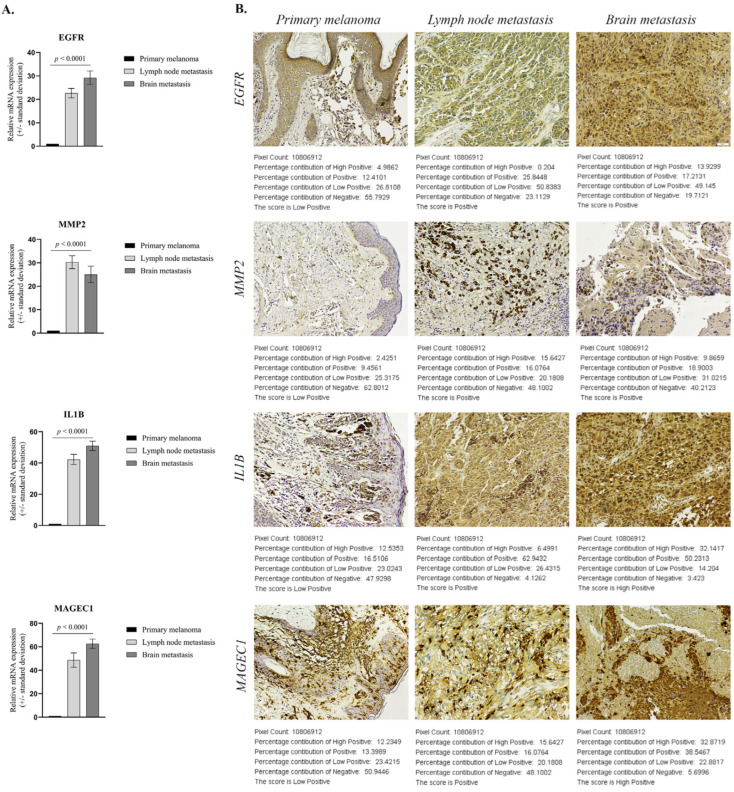
Expression of select markers in patient samples (n = 10/tumor stage): (**A**) Statistically significant differential expression of *EGFR*, *MMP2*, *IL1B* and *MAGEC1* in patient FFPE samples (*p* < 0.0001); (**B**) Representative immunohistochemistry images for protein expression of EGFR, MMP2, IL1B, MAGEC1 (scale bar 50µm) and score determined by IHC Profiler. The log given below the stained images shows the accurate percentage of the pixels present in each zone of pixel intensity and the respective computed score (Low Positive in PM and either Positive or Highly Positive in metastases).

Validation studies were performed using qPCR and standard immunohistochemistry. From the genes analyzed, representative markers (*EGFR*, *MMP2*, *IL1B* and *MAGEC1*) were selected and assayed on Formalin-Fixed Paraffin-Embedded (FFPE) tissue sections from PM, LNM and BM patients (Figure 6).

#### 2.2.1. Validation of Select Genes’ Expression between Primary and Metastatic Melanoma

To confirm the reliability and accuracy of the results obtained through the above analysis, we verified the mRNA level of select genes on distinct stages of melanoma progression FFPE samples. As shown in Figure 6A, *EGFR*, *MMP2*, *IL1B* and *MAGEC1* gene expression levels showed consistent up-regulation (in the sense of the immunohistochemical expression of the homologous genes), with statistically significant increases of more than 20-fold (*p* < 0.0001) in metastatic sites (n = 10 each) compared to primary melanoma (n = 10). Overexpression of *EGFR*, *IL1B* and *MAGEC1* was higher in BM than LNM specimens, while *MMP2* was better expressed in LNM than BM samples.

#### 2.2.2. Validation of Protein Expression of Select Genes in Primary and Metastatic Melanoma Specimens

To further confirm the protein expression levels of these select genes in metastasis development, sections from FFPE samples were analyzed through IHC analysis and a corresponding score was generated with IHC profiler, an automated digital IHC image analysis algorithm, for an unbiased, quantitative assessment of antibody staining intensity in tissue sections [12]. As shown in Figure 6B, the protein expression of these four markers was significantly up-regulated in metastatic melanoma samples compared with the primary melanoma specimens. Quantitative analysis of digital IHC images showed that EGFR and MMP2 were Positive (+2) in LNM and BM, compared to the Low Positive (+1) corresponding scoring log found in PM samples, while IL1B and MAGEC1 had a High Positive (+3) score in metastases.

## 3. Discussion

Cutaneous melanoma is a clinically heterogeneous disease with an unpredictable response to treatment, whose process of malignant transformation, progression and metastasis continues to be poorly understood. So far, insight into the genes and non-coding RNA (ncRNA) molecules involved in these processes has been gathered through the use of human cancer expression profiling [2]. Thus, in this study, we utilized qPCR arrays, the most reliable tools for the parallel quantitative analysis of gene expression signatures [13] and a variety of molecular techniques to comparatively evaluate primary (MelJuSo) and metastatic melanoma cells derived from the lymph node (MNT-1) and brain (VMM1), at gene and miRNA levels, in order to identify potential markers of prognostic value that could also be used as therapeutic targets. A number of statistical software packages, including the GeneGlobe analysis program, were utilized to compare primary tumor cells to metastatic melanoma cell lines. After the expression levels of putative oncogenes and tumor suppressor genes were analyzed, immunofluorescence staining coupled with confocal microscopy was performed on select genes, potential metastasis drivers due to their high level of expression in both LNM and BM cell lines, in order to confirm the results at the protein level. Furthermore, the few promising genes (*EGFR*, *MMP2*, *IL1B* and *MAGEC1*), found to be distinctly and consistently dysregulated between primary and metastatic cells, were investigated in FFPE patient samples by qPCR and IHC analysis.

Gene expression profiling identified around 200 genes that showed significant differential expression in metastatic cell lines compared to primary tumor cells, with 27 of the most statistically significant ones being further graphically represented. Most differences reflected increased gene expression in MNT-1 and VMM1 relative to MelJuSo cells. Reduced gene expression in metastatic cell lines was less frequent and less dramatic. Genes significantly increased or decreased in LNM- and BM-derived cells were active in cell cycle regulation, proliferation, apoptosis, cell motility, inflammation, angiogenesis and immune response modulation. From the list of genes that showed relatively increased expression in metastatic cells, immunohistochemistry targeted to EGFR, MMP2, IL1B and MAGEC1 epitope expression showed strong positive cytoplasmic staining in the tumor cells of LNM and BM patient samples (Positive or High Positive score), while melanoma cells at the primary site had a low expression of these markers (Low Positive), results which were corroborated at gene expression level (more than 20-fold change).

We found that metastatic melanoma cell lines express significantly higher levels of many genes, including *BCL-2*, *IGF-1*, *EGFR*, *FGFR2*, *MMP1*, *MMP2*, *MMP3*, *RHOU*, *TNF*, *IL1B*, *FLT-1* and *MAGEC1*, when compared to the PM cell line, although some of them were oppositely expressed between MNT-1 and VMM1. These results are in accordance with other studies published so far. For instance, up-regulation of *BCL-2* has been observed in most melanomas, being correlated with a poor prognosis for patients [14]. At the same time, *IGF-1* was found to contribute to the expansion of melanoma-initiating cells through an epithelial-mesenchymal transition process [15]. Although the role of *EGFR* has been established in a range of neoplasms, in melanoma the results are conflicting. However, our data align with the ones where overexpression of *EGFR* was linked to metastatic progression and worse prognosis, such as the study of Xie et al., who defined *EGFR* as a hub gene in an experiment that screened the differentially expressed genes between melanoma metastases and PM using the GEO2R tool [16]. Further, *FGFR2* is frequently found up-regulated in melanoma, its expression level increasing with tumor progression, and is suggested to be involved in mediating cell proliferation, survival and migration. Through FGFR signaling, tumor cells can develop the ability to influence neighboring cells, such as endothelial cells, fibroblasts or other malignant melanocytes found within the tumor niche, in order to promote cell survival and even angiogenesis, which can ultimately contribute to the appearance of resistance to therapy [17].

During melanoma progression, MMPs facilitate invasion and metastasis and participate as regulators of tumor cells’ proliferation and apoptosis, also being capable of influencing immune-resistance and angiogenesis. In our study, we found several MMP molecules to be significantly overexpressed in metastatic cell lines compared to primary tumor cells (e.g., *MMP1*, *MMP2*, *MMP3*, *MMP9*), with a dramatic increase registered for *MMP2*. This gene in particular has been suggested as a prognostic biomarker, as patients with strong expression levels had significantly poorer survival compared to those with negative-to-moderate *MMP2* expression in a study conducted on tumor samples representing various stages of melanoma progression [18]. Another particular event we found was the overexpression of *RHOU* in metastatic cell lines compared to primary tumor cells, and this is of interest because it encodes for a Rho GTPase involved in cell motility through lamellipodia formation, highlighting its potential role in melanoma invasion [19].

Various other molecules contribute to melanoma progression, including pro-inflammatory cytokines, some of which we discovered to be aberrantly regulated in our expression profiling. Current available data suggest that TNF-α and interleukins IL1B, IL-6, IL-8 and IL-18 can modulate the proliferation, survival and migration of cutaneous melanoma cells. PM samples express low levels of these markers, while more advanced stages show higher expression levels. In addition, a correlation between *TNF-α* overexpression and an increased risk of tumor development in vivo has been observed [20,21]. Moreover, IL1B and its family member IL-18 were demonstrated to promote invasion and angiogenesis through the activation of NF-κB, which triggers the secretion of VEGF from tumor cells, induces their proliferation and migration, prevents apoptosis, and may also induce PD1-dependent immunosuppression of natural killer (NK) cells [22]. We also observed a high expression of angiogenesis-related markers in metastatic melanoma cell lines. *FLT1*, a gene that encodes for vascular endothelial growth factor receptor 1 (VEGFR1) was up-regulated in both MNT-1 and VMM1, while *VEGFA* showed significantly higher expression only in VMM1, compared to MelJuSo. Their association with an aggressive angiogenic phenotype in malignant melanoma and poor prognosis has been reported [23].

Finally, multiple markers involved in immune response modulation were highlighted, among them *MAGEC1*, with a significant overexpression in different metastatic stages compared to PM samples. Melanoma-associated antigen C1 (MAGEC1) is a potential target in immunotherapy because its expression is present only in tumor cells. A recent study investigated *MAGEC1* expression in primary and metastatic melanoma, reporting the predisposition of *MAGEC1*-positive patients to metastasize, with a significantly higher number of lymph node metastases [24]. Additionally, some researchers found that metastatic melanomas express higher levels of *MAGE* compared to primary stage, suggesting this gene to be a strong candidate for driving metastatic progression [25,26]. *KIR2DL3* and *HLA-DRB1* were also up-regulated in MNT-1 and VMM1 cell lines, respectively. Concerning *HLA-DRB1*, this marker plays an important role in immune antigen presentation and its overexpression has been correlated with increased risk of melanoma recurrence [27]. However, results are inconsistent, with some suggesting *HLA-DRB1* to be positively correlated with the survival, prognosis and tumor microenvironment remodeling of melanoma patients [28]. Meanwhile, higher expression of *KIR2DL3*, a receptor that inhibits NK cell activation, has been reported in stage III and IV melanoma patients compared to stage I melanoma, and several clinical trials are underway to test inhibitors, including Lirilumab and ASP98374 [29].

Regarding the profiling of ncRNAs, differentially expressed miRNAs were found between PM, LNM and BM cell lines, but the completely opposite regulation between the two different types of metastases was remarkable. Thus, miR-129-2-3p and let-7e-5p were overexpressed in MNT-1 but down-regulated in VMM1, while miR-100-5p, miR-125b-5p, let-7a-5p, let-7d-5p, let-7f-5p and let-7g-5p registered a much higher expression level in VMM1, but decreased significantly in MNT-1 compared to MelJuSo cells. Data currently available reveal that miR-100-5p and miR-125b-5p can promote tumor cell proliferation and invasion, at the same time being related to survival time as they can negatively affect patient response to treatment with BRAF inhibitors such as vemurafenib [6,9,30]. Additionally, together with let-7e-5p, they can influence the differentiation and polarization of monocytes towards the immunosuppressive phenotype (MDSCs), indicating a potential negative effect on immunotherapy [31]. Let-7 family members are known to be involved in cell cycle regulation, proliferation, apoptosis, differentiation and EMT transition. They are generally thought to act as tumor suppressors, regulating the expression of oncogenes (e.g., *RAS*) to reduce cancer aggressiveness; however, the situation is uncertain due to conflicting data revealing both their down-regulation and up-regulation in different types of cancer. It has been reported that in rare situations let-7 acts as an oncogene, increasing cancer migration, invasion, chemoresistance, and the expression of genes associated with progression and metastasis [32,33]. For example, Ma et al. demonstrated that let-7e is overexpressed and positively affects migration and invasion of esophageal squamous cell carcinoma cells, possibly via targeting *ARID3a* [34]. Considering *ARID3a* negatively correlates with pluripotency, its decrease could contribute to stemness [35]. Additionally, let-7f and let-7e have been shown to be up-regulated in lingual squamous carcinoma, while let-7c, let-7d, and let-7f are up-regulated in aggressive tumors relative to non-aggressive ones [36]. These results indicate the complexity of the relationship between let-7 and cancer cell aggressiveness, suggesting the fact that the actions of any miRNAs are context dependent. Finally, information regarding the role of miR-129-2-3p in cancer progression is scarce. One study reported that miR-129 acts as a tumor suppressor, and its overexpression leads to decreased cell proliferation and viability of melanoma cells resistant to vemurafenib, possibly by modulating SOX4 expression [37]. Nevertheless, the mechanisms underlying its deregulation and contribution to melanomagenesis remain elusive. Bioinformatic analysis performed using the miRNet 2.0 algorithm led to the identification of numerous target genes, including *BCL-2*, *EGFR*, *NRAS*, *KRAS*, *RAF*, *IGF1*, *FGFR2*, *TNF*, *IL-6*, *MMP2* and *MMP9*, whose altered gene expression was also revealed by qPCR array.

Interestingly, most of the evaluated genes and miRNAs were differentially expressed between the two distinct types of metastatic melanoma cells. Thus, the results obtained in this study seem to not align with the Clark model that depicts a stepwise evolution of morphological abnormalities accompanying the pathological process, which drive melanocytes through a linear progression towards metastatic stages. Instead, the results seem to favor the parallel progression model, which suggests that metastatic melanoma can evolve from any tumor phase, without necessarily passing through all of them, as the dissemination of precancerous or malignant cells to distant sites often occurs from early transformed lesions, and the acquisition of important molecular alterations is not exclusively confined to the primary lesion, but rather takes place at the metastatic sites in a specific manner [38,39,40].

## 4. Materials and Methods

### 4.1. Melanoma Cell Lines and Human Tissue Samples

Cell lines included primary melanoma (MelJuSo), lymph node metastasis (MNT-1) and cerebral metastasis (VMM1) of human origin, which were kindly provided by partner institutions from the 61PCCDI-PATHDERM project (National Institute of Pathology ”Victor Babes” and Institute of Biochemistry of the Romanian Academy). Melanoma MelJuSo cells were cultured in RPMI-1640 medium (Sigma Aldrich Co., Steinheim, Germany) supplemented with 10% fetal bovine serum (FBS) and 1% antibiotic antimycotic at 37 °C in a 5% CO_2_ incubator, while metastatic cells were additionally supplemented with 10% FBS and 1% from commercial solutions of glutamine, sodium pyruvate and non-essential amino acids, purchased from Thermo Fisher Scientific (Waltham, MA, USA). The medium was changed every two–three days. Patient samples derived from Formalin-Fixed Paraffin-Embedded (FFPE) tissue blocks containing excisional biopsies or surgical specimens histopathologically confirmed as melanomas were provided by the Emergency University Hospital Bucharest, Romania. Tissue samples from 30 patients, both male and female, with cutaneous melanoma, lymph node or brain metastases were collected.

### 4.2. Gene Expression Array and Data Analysis

A large-scale expression screening of 252 genes was assessed using the ExProfile™ Human Gene qPCR Array (GeneCopoeia Inc., Rockville, MD, USA) between primary and metastatic melanoma cell lines. Briefly, total RNA from melanoma cells was isolated using Trizol reagent (Thermo Fisher Scientific) according to the manufacturer’s protocol, and tested for integrity on a BioAnalyzer 2100 (Agilent Technologies, Boblingen, Germany). One microgram of total cellular RNA was reverse transcribed to the corresponding cDNA using an iScript cDNA Synthesis kit (BioRad, Hercules, CA, USA). Subsequently, we used the All-in-One qPCR Mix Kit (GeneCopoeia) and the Viia7 Real-Time PCR System (Applied Biosystems, Waltham, MA, USA) for gene expression evaluation. Data analysis was performed using the Analysis Tool available from the manufacturer (https://www.genecopoeia.com/product/gene-qpcr-array/#Analysis_Tool, accessed on 9 October 2022), mainly based on the ΔΔCt method [41], with normalization of the raw data to several housekeeping genes (*GAPDH*, *ACTB*, *B2M*, *RPL13A*, *HPRT1*, *RN18S1*). Corresponding heatmaps were generated through the use of GeneGlobe Data Analysis Center (https://geneglobe.qiagen.com/ro/analyze, accessed on 11 October 2022), the Real-Time PCR module for gene, and miRNA expression (Qiagen, Hilden, Germany), and statistical analysis for select genes was performed with GraphPad Prism software 9.0 (GraphPad Software Inc., San Diego, CA, USA) using an analysis of variance (ANOVA), with *p*-values adjusted for multiple comparisons (Bonferroni correction). Data are expressed as the mean ± standard deviation (SD); significant statistical differences were considered for *p* < 0.05.

### 4.3. microRNA Expression Profile and Integrative Bioinformatic Analyses

Primary and metastatic cell cultures were subjected to a comparative evaluation of the expression level of 84 miRNA molecules potentially involved in melanoma invasion and metastasis, in biological triplicate, using miProfile™ human miRNA qPCR arrays (GeneCopoeia) and a Viia7 Real-Time PCR System (Applied Biosystems). Data analysis was performed using the Data Analysis System from the manufacturer (https://www.genecopoeia.com/product/qpcr/analyse/, accessed on 9 October 2022), based on the 2^−∆∆Ct^ method, with normalization of the raw data to several small RNA molecules as an endogenous reference (U6, SNORD44, SNORD47, SNORD48, SNORD49A, SNORD68). Corresponding heatmaps were generated through the use of GeneGlobe (Qiagen), and statistical analysis for the miRNAs of interest was performed using GraphPad Prism software (version 9.0), ANOVA algorithm and Bonferroni correction. Data are expressed as the mean ± SD; *p* < 0.05 was considered statistically significant. Functional analysis and visual exploration of miRNA-target interactions in a network context was determined using miRNet 2.0. platform [11].

### 4.4. Protein Expression by Immunolabeling and Confocal Microscopy Analysis

Protein expression levels of selected markers were revealed in cell cultures depicting distinct melanoma progression stages through immunofluorescence staining and confocal microscopy observation. Cells were fixed with a 4% paraformaldehyde solution (Sigma Aldrich) for 1 h, after which the cell membrane was permeabilized using 0.1% Triton X-100 (Sigma Aldrich) and 1.2% bovine serum albumin (BSA, Sigma Aldrich) solution for 30 min. Samples were then incubated overnight at 4 °C with EGFR antibody (rabbit monoclonal antibody, Cell Signaling, Danvers, MA, USA) diluted 1:100, MMP2 (rabbit monoclonal antibody, Cell Signaling) diluted 1:800, IL1B (rabbit monoclonal antibody, Cell Signaling) diluted 1:500 and MAGEC1 (mouse monoclonal antibody, Santa Cruz Biotechnology, Dallas, TX, USA) diluted 1:200. Afterwards, they were incubated for 2 h at RT, in the dark, with anti-mouse Alexa Fluor 546 and anti-rabbit Alexa Fluor 647 secondary antibodies (Invitrogen, Waltham, MA, USA). Finally, actin filaments were stained during 1 h of incubation with phalloidin-FITC (Sigma Aldrich) and cell nuclei with Hoechst 33342 for 5 min (Thermo Fisher Scientific). A Nikon AX R Eclipse Ti2-E Confocal Microscope system was used for visualization and the images were analyzed using the corresponding Nikon Nis Elements software (Nikon Instruments Inc., version 5.21 64-bit, Melville, NY, USA). Quantification of fluorescence intensity was performed with ImageJ software (version 1.x bundled with 64-bit Java 8), while GraphPad Prism (version 9.0) was used for graphical representation and statistical analysis (ANOVA algorithm and Bonferroni correction). Data are expressed as mean ± SD for n = 10 fields of view/melanoma cell line; *p* < 0.05 was considered statistically significant.

### 4.5. Gene Expression by Quantitative Real-Time PCR (qRT-PCR) and Statistics

A qRT-PCR on human melanoma specimens (n = 30, 10/tumor stage) was performed to confirm the results of the expression array data. Total RNA was isolated from FFPE tissue samples using PureLink™ FFPE RNA Isolation Kit (Thermo Fisher Scientific) per manufacturer’s protocol. iScript™ cDNA Synthesis Kit (BioRad) was employed for complementary DNA synthesis, while qPCR reactions were performed using Viia7 machine (Applied Biosystems), Forget-Me-Not™ EvaGreen^®^ qPCR Master Mix (Biotium, Fremont, CA, USA) and specific primer sequences. An expression evaluation of selected target genes (*EGFR*, *MMP2*, *IL1B* and *MAGEC1*) was run in triplicate and values were normalized to *Actin* and *GAPDH* reference genes. Statistical analysis was performed with GraphPad Prism software (version 9.0), using the one-way ANOVA algorithm and Bonferroni correction. Data are expressed as the mean ± SD; *p* < 0.05 was considered statistically significant.

### 4.6. Immunohistochemistry (IHC) and Automated Scoring

Sections from each FFPE patient sample were cut using a microtome, then mounted on slides coated with a suitable tissue adhesive. Samples were de-paraffinized in Dewax solution (Leica Biosystems, Wetzlar, Germany) and re-hydrated in graded alcohols (100%, 95%, 70% ethanol). Antigen retrieval was performed for 20 min in a water bath at 95 °C using 1× Target Retrieval Solution (Dako, Agilent Technologies, Santa Clara, CA, USA). The sections were then stained using Novolink Max Polymer Detection System (Novocastra, Leica Biosystems) according to the manufacturer’s instructions. Samples were evaluated for the expression of EGFR (diluted 1:50), MMP2 (diluted 1:200), IL1B (diluted 1:800) and MAGEC1 (diluted 1:300). IHC Select^®^ 20× TBS Rinse Buffer (Merck Millipore, Burlington, MA, USA) was used for washing slides according to the instructions provided. Ultimately, slides were dehydrated in Unyhol and Unyhol Plus solutions (Bio-Optica, Milano, Italy), cleared with BioClear solution (BioGnost, Zagreb, Croatia), and permanently mounted using CC/Mount™ medium (Sigma-Aldrich). Images were captured at 20× objective lenses with Nikon Eclipse Si optical microscope and corresponding Mshot Digital Imaging Software V1.1.6 (Nikon Instruments Inc.). IHC Profiler, an open-source plugin, was used for the quantitative evaluation and automated scoring of immunohistochemistry images [12].

## 5. Conclusions

In this study, the comparative expression profiling of primary and metastatic melanoma cell lines has resulted in the identification of several genes and miRNAs that might be centrally involved in the progression and metastatic potential of this type of skin cancer. A number of the identified markers were previously found in melanoma, including established cancer-related genes, such as *BRAF* or *RAS*. However, the multi-level analysis highlighted a short list of candidate genes (*EGFR*, *MMP2*, *IL1B*, *MAGEC1*) and miRNAs (miR-100, miR-125b, miR-129, several let-7 family members) that may have a causative role in the malignant progression of melanoma, and which could represent promising targets for future investigation. Our results may contribute to the improved understanding of the metastatic process, with potential heavy implications for prognostic marker identification and targeted therapy development.

## Figures and Tables

**Table 1:** KEGG database analysis of genes associated with melanoma metastasis targeted by the miRNAs of interest.

Term	Count	*p* Value	Notable Genes
Pathways in cancer	22	1.44 × 10^−6^	*CDKN2A*, *BCL-2*, *EGFR*, *NRAS*, *KRAS*, *IL6*, *IGF1*, *IGF1R*, *FGFR2*, *MMP2*, *MMP9*, *PDGFB*, *STK4*, *E2F2*, *E2F3*
Cell cycle	12	2.26 × 10^−5^	*CCND1*, *CDKN1A*, *MYC*, *CDKN2A*, *CREBBP*, *CDK6*
Melanoma	9	2.1 × 10^−5^	*CCND1*, *CDKN1A*, *CDKN2A*, *EGFR*, *KRAS*, *NRAS*, *IGF1*, *IGF1R*, *RAF1*
MAPK signaling pathway	8	2.67 × 10^−3^	*EGFR*, *KRAS*, *NRAS*, *MYC*, *FGFR2*, *TNF*, *PDGFB*, *TGFBR1*
Apoptosis	3	3 × 10^−3^	*BCL-2*, *CASP3*, *XIAP*
VEGF signaling pathway	2	5.28 × 10^−3^	*KRAS*, *PLCG2*

## Data Availability

Not applicable.

## Supplementary Materials

The supporting information can be downloaded at: https://www.mdpi.com/article/10.3390/ijms24076565/s1.
