# Supplementary material for: Comparative Expression Profiling Reveals Molecular Markers Involved in the Progression of Cutaneous Melanoma towards Metastasis
Source: Int J Mol Sci. 2023 Mar 31;24(7):6565. doi: 10.3390/ijms24076565 (PMC10095431; doi:10.3390/ijms24076565)
Supplement: Supplementary file 1 [file ijms-24-06565-s001.zip › ijms-2234477-supplementary-Figure S1.pdf]

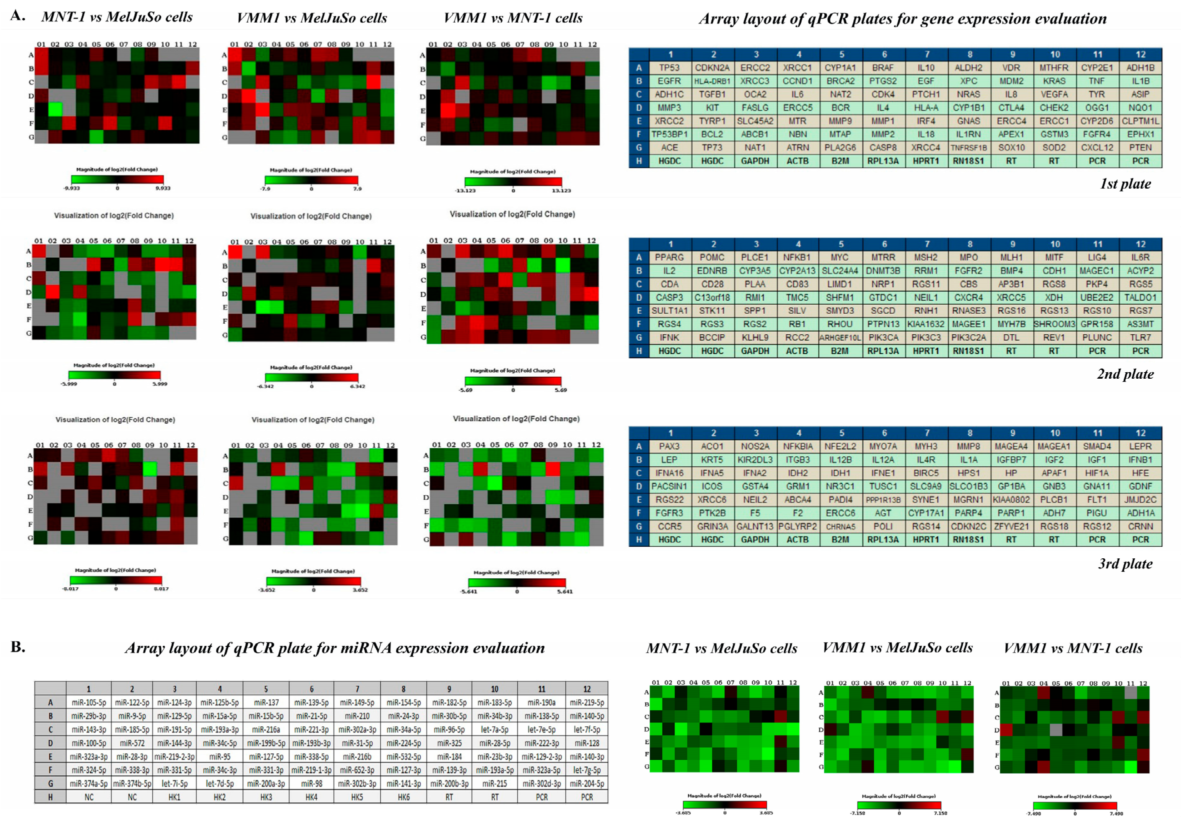

Figure S1. (**A**) Heatmaps corresponding to the expression of 252 genes evaluated using qPCR array (n = 3, biological replicates), which showcase the differential expression between LNM and PM cell lines (first lane); BM and PM cell lines (second lane); BM and LNM cell lines (third lane). Red stands for up-regulation, green represents down-regulation, black reveals no significant differences between evaluated genes, while grey means that the expression of the genes was insignificant in our experimental and statistical analysis. The array layout corresponding to each qPCR plate is presented on the right side; (**B**) Heatmaps corresponding to the expression of 84 miRNAs evaluated using qPCR array (n = 3, biological replicates), which showcase the differential expression between: LNM and PM cells (first heatmap), BM and PM cell lines (second heatmap), BM and LNM cell lines (third heatmap). The corresponding array layout is presented on the left side.
