# Supplementary material for: Comparative Expression Profiling Reveals Molecular Markers Involved in the Progression of Cutaneous Melanoma towards Metastasis
Source: Int J Mol Sci. 2023 Mar 31;24(7):6565. doi: 10.3390/ijms24076565 (PMC10095431; doi:10.3390/ijms24076565)
Supplement: Supplementary file 1 [file ijms-24-06565-s001.zip › ijms-2234477-supplementary-Table S1.pdf]

Table S1. (A) List of differentially expressed genes in MNT-1 cell line compared to MelJuSo cells

Gene	Fold Difference	T-TEST	Fold Up- or Down-Regulation
	Test Sample /Control Sample	p-value	Test Sample /Control Sample
MITF	3.616512996	5.96207E-06	3.616512996
BRAF	3.945386556	6.47205E-05	3.945386556
KRAS	3.224703169	0.000926793	3.224703169
NRAS	0.632658497	0.219456341	-1.58063158
BCL2	26.01311481	1.81238E-09	26.01311481
EGF	9.322183878	7.37221E-05	9.322183878
EGFR	53.13047417	4.36894E-10	53.13047417
IGF1	16.25151812	5.53056E-06	16.25151812
IGF2	16.21398848	0.001769113	16.21398848
FGFR2	45.05664074	1.66062E-05	45.05664074
FGFR4	8.127059237	6.55312E-13	8.127059237
MMP1	18.11775452	2.72919E-13	18.11775452
MMP2	21.11455513	1.01272E-08	21.11455513
MMP3	19.19287611	1.40483E-14	19.19287611

MMP9	23.50398292	1.84565E-09	23.50398292
RHOU	2.086619647	9.97469E-05	2.086619647
TNF	38.99365328	4.16185E-05	38.99365328
IL1B	4.602759836	4.4044E-05	4.602759836
IL6	2.287597111	0.00589901	2.287597111
IL8	0.433218685	0.029558976	-2.308303021
IL18	5.578324378	3.09294E-10	5.578324378
VEGFA	0.404113825	0.005662779	-2.47455033
FLT1	3.888025889	1.29965E-05	3.888025889
HLA-A	0.353431266	0.001326601	-2.829404458
HLA-DRB1	0.04984184	0.000434932	-20.0634648
KIR2DL3	8.037990732	1.07896E-05	8.037990732
MAGEC1	112.6470112	8.74115E-09	112.6470112

Table S1. (B) List of differentially expressed genes in VMM1 cell line compared to MelJuSo cells

Gene	Fold Difference	T-TEST	Fold Up- or Down-Regulation
	Test Sample /Control Sample	p-value	Test Sample /Control Sample
MITF	0.044209478	0.000279133	-22.61958409
BRAF	1.068188792	0.538420589	1.068188792
KRAS	0.645952493	0.001746577	-1.548101464
NRAS	1.6492757	0.036402799	1.6492757
BCL2	0.229118713	2.49526E-08	-4.364549651
EGF	1.747347993	7.49102E-05	1.747347993
EGFR	110.4946897	8.74348E-09	110.4946897
IGF1	3.239658218	9.00796E-06	3.239658218
IGF2	4.178064815	6.55415E-05	4.178064815
FGFR2	0.335449361	0.002619528	-2.98107588
FGFR4	0.864277399	0.001766503	-1.157035925
MMP1	7.76740089	1.58598E-08	7.76740089
MMP2	150.7346076	6.0105E-06	150.7346076
MMP3	9.723653021	3.81826E-08	9.723653021
MMP9	1.303597961	6.83169E-06	1.303597961
RHOU	8.374681748	6.14694E-08	8.374681748
TNF	1.010566999	0.850413048	1.010566999
IL1B	33.04055349	1.13641E-06	33.04055349
IL6	24.95228325	8.96535E-06	24.95228325
IL8	65.0060109	1.63791E-09	65.0060109
IL18	0.27071224	0.019784861	-3.693959308
VEGFA	14.41501494	2.07115E-09	14.41501494
FLT1	5.149262301	3.60111E-05	5.149262301
HLA-A	0.243754537	0.002831543	-4.102487743
HLA-DRB1	2.013214168	0.01303085	2.013214168
KIR2DL3	0.913726118	0.212234892	-1.094419849
MAGEC1	18.85009872	1.06461E-08	18.85009872

Table S1. (C) List of differentially expressed genes in VMM1 cell line compared to MNT-1 cells

Gene	Fold Difference	T-TEST	Fold Up- or Down-Regulation
	Test Sample /Control Sample	p-value	Test Sample /Control Sample
MITF	0.012382963	9.10223E-05	-80.7561169
BRAF	0.27074376	8.93382E-05	-3.693529259
KRAS	0.200313784	3.26927E-05	-4.992167698
NRAS	2.511182617	3.25346E-06	2.511182617
BCL2	0.008807815	1.59437E-09	-113.5355312
EGF	0.187426826	2.08538E-05	-5.335415543
EGFR	2.079685745	1.28014E-07	2.079685745
IGF1	0.199344959	1.09857E-05	-5.01642983
IGF2	0.25768273	0.00421895	-3.880741252
FGFR2	0.006019387	1.55602E-05	-166.1298624
FGFR4	0.10634565	2.16271E-10	-9.403299503
MMP1	0.428717636	2.74791E-09	-2.332537586
MMP2	7.138895736	1.06877E-05	7.138895736
MMP3	0.506628239	2.73766E-08	-1.973833915
MMP9	0.055462853	1.96095E-09	-18.03008567
RHOU	3.493968786	0.000210052	3.493968786
TNF	0.025916192	4.16407E-05	-38.58591595
IL1B	7.178422222	2.10491E-06	7.178422222
IL6	10.90763891	1.31717E-05	10.90763891
IL8	150.0535713	2.92252E-09	150.0535713
IL18	0.048082871	8.34047E-06	-20.79742683
VEGFA	35.67067999	3.25671E-08	35.67067999
FLT1	1.373028348	0.000706324	1.373028348
HLA-A	0.689680173	0.334892339	-1.449947437
HLA-DRB1	40.39205161	0.000892407	40.39205161
KIR2DL3	0.113675936	1.12158E-05	-8.796936607
MAGEC1	0.167337762	1.85333E-08	-5.975937468

Table S1. (D) List of differentially expressed miRNAs in MNT-1 cell line compared to MelJuSo cells

miRNA	Fold Difference	T-TEST	Fold Up- or Down-Regulation
	Test Sample /Control Sample	p-value	Test Sample /Control Sample
hsa-miR-100-5p	0.121694358	2.88115E-10	-8.217307812
hsa-miR-125b-5p	0.342323606	8.71533E-09	-2.92121251
hsa-miR-129-2-3p	1.483906072	1.4319E-07	1.483906072
hsa-let-7a-5p	0.895049904	4.03476E-06	-1.117256139
hsa-let-7d-5p	0.204381239	4.6221E-09	-4.89281699
hsa-let-7e-5p	2.326348252	8.11493E-08	2.326348252
hsa-let-7f-5p	0.566743592	6.10291E-06	-1.764466355
hsa-let-7g-5p	0.430972437	2.06288E-08	-2.320334005

Table S1. (E) List of differentially expressed miRNAs in VMM1 cell line compared to MelJuSo cells

miRNA	Fold Difference	T-TEST	Fold Up- or Down-Regulation
	Test Sample /Control Sample	p-value	Test Sample /Control Sample
hsa-miR-100-5p	2.147511925	2.17E-08	2.147511925
hsa-miR-125b-5p	2.753624984	1.2E-08	2.753624984
hsa-miR-129-2-3p	0.195205686	3.4E-11	-5.122801591
hsa-let-7a-5p	6.194554167	3.17E-09	6.194554167
hsa-let-7d-5p	1.790464814	6.97E-08	1.790464814
hsa-let-7e-5p	0.583983683	1.68E-06	-1.712376611
hsa-let-7f-5p	4.291057283	1.2E-08	4.291057283
hsa-let-7g-5p	3.113061923	8.82E-09	3.113061923

Table S1. (F) List of differentially expressed miRNAs in VMM1 cell line compared to MNT-1 cells

miRNA	Fold Difference	T-TEST	Fold Up- or Down-Regulation
	Test Sample /Control Sample	p-value	Test Sample /Control Sample
hsa-miR-100-5p	17.64676652	1.94E-09	17.64676652
hsa-miR-125b-5p	8.043923752	2.64E-09	8.043923752
hsa-miR-129-2-3p	0.131548546	2.8E-09	-7.601756385
hsa-let-7a-5p	6.920903672	2.9E-09	6.920903672
hsa-let-7d-5p	8.760416662	2.65E-09	8.760416662
hsa-let-7e-5p	0.251030207	9.99E-09	-3.983584336
hsa-let-7f-5p	7.571426203	2.72E-09	7.571426203
hsa-let-7g-5p	7.223343438	2.9E-09	7.223343438
